# Pertuzumab combined with trastuzumab compared to trastuzumab in the treatment of HER2-positive breast cancer: A systematic review and meta-analysis of randomized controlled trials

**DOI:** 10.3389/fonc.2022.894861

**Published:** 2022-09-28

**Authors:** Xiaoyun Liu, Yingying Fang, Yinjuan Li, Yan Li, Lu Qi, Xinghe Wang

**Affiliations:** Department of Phase I Clinical Trail Center, Beijing Shijitan Hospital, Capital Medical University, Beijing, China

**Keywords:** breast cancer, HER2-positive, pertuzumab, trastuzumab, meta-analysis

## Abstract

**Objective:**

Although dual anti-HER2 therapy, namely, pertuzumab plus trastuzumab, has shown promising results in patients with HER2-positive breast cancer (BC), it is still unclear whether dual therapy will increase adverse effects (AEs) while ensuring the efficacy compared with trastuzumab monotherapy. We conducted a systematic review and meta-analysis to compare the efficacy and safety of combined therapy with monotherapy.

**Methods:**

A systematic search was performed to identify eligible randomized controlled trials (RCTs) that evaluated the administration of dual anti-HER2 therapy [pertuzumab plus trastuzumab or trastuzumab emtansine (T-DM1)] versus monotherapy (trastuzumab or T-DM1). The primary endpoints were overall survival (OS) and progression-free survival (PFS).

**Results:**

Fourteen RCTs (8,378 patients) were identified. Compared to monotherapy, dual therapy significantly improved the OS (HR = 0.77, 95% CI: 0.59–0.99) and PFS (HR = 0.74, 95% CI: 0.63–0.86) in advanced BC. In neoadjuvant therapy, dual blockade has a higher ORR rate than monotherapy. Grade 3 or higher febrile neutropenia, diarrhea, and anemia as well as heart failure were more frequently reported in dual therapy compared to monotherapy. No significant difference in serious AEs was observed between the two groups. In the subgroup analysis, compared to single-target therapy, dual-target therapy has higher OS and PFS rates in Asian patients with advanced therapy; however, total grade ≥3 AEs and serious AEs were significantly higher in the dual group in Asian patients.

**Conclusions:**

Our study confirms that the combination of pertuzumab and trastuzumab therapy could substantially improve the outcome of patients with HER2-positive breast cancer and was well tolerated compared to trastuzumab monotherapy.

## Introduction

In 2020, breast cancer was the fourth leading cause of cancer-related deaths among women in China and ranked first in the incidence of female cancer (1). Approximately 20% of breast cancers strongly overexpressed human epidermal growth factor receptor 2 (HER2), which has historically been associated with a poor prognosis, an aggressive phenotype, and a shorter overall survival (OS) (2). Trastuzumab, a recombinant humanized monoclonal antibody targeting HER2, combined with chemotherapy increased response rates and time to progression. However, the majority of cancers that initially respond to trastuzumab begin to progress again within 1 year (3). Moreover, the cardiotoxicity rate in the trastuzumab arm (4.0%) was higher than in the non-trastuzumab arm (1.3%) (4).

Pertuzumab, another monoclonal HER2 antibody, binds to a different region of trastuzumab and blocks the dimerization of HER2 with other HER family members such as HER3. Previous studies proved that the combination of trastuzumab and pertuzumab demonstrated a strongly enhanced antitumor effect combined as compared with either agent alone in preclinical studies (5). Pertuzumab was approved by the FDA for use in combination with trastuzumab and chemotherapy for the advanced, neoadjuvant, and adjuvant treatment of patients with HER2-positive breast cancer (6, 7). Although they generally are well tolerated, HER2-targeted therapies are associated with cardiotoxicity such as an asymptomatic decrease in the left ventricular ejection fraction (LVEF) (8).

Therefore, it is still unclear whether the combination of pertuzumab and trastuzumab does not increase the incidence of adverse effects (AEs) while enhancing the antitumor effect, compared with trastuzumab single-agent therapy. We conducted an up-to-date systematic review and meta-analysis of randomized controlled trials (RCTs) on pertuzumab plus trastuzumab (or ado-trastuzumab emtansine, T-DM1) dual anti-HER2 therapy compared with trastuzumab or T-DM1 monotherapy in patients with HER2-positive breast cancer. Due to the effect of conjugated trastuzumab, i.e., T-DM1, which is expected to be greater than the original trastuzumab, we divided pertuzumab plus trastuzumab or T-DM1 into two groups.

## Material and methods

### Study design, patients, comparison, and outcome

We quantitatively summarized data on the efficacy and safety of pertuzumab from RCTs. Patients were diagnosed with HER2-positive breast cancer. The primary endpoints were progression-free survival (PFS) and OS. The secondary endpoints included pathologic complete response (pCR), partial response (PR), complete response (CR), and objective response rate (ORR). The safety endpoints were adverse events (AEs) including cardiotoxicities, serious AEs, any-grade AEs, and so on. The risk estimates were pooled by comparing the pertuzumab plus trastuzumab (or pertuzumab plus T-DM1) group with or without chemotherapy versus the trastuzumab (or T-DM1) monotherapy group with or without chemotherapy.

### Search strategy and selection criteria

This systematic review adhered to the Preferred Reporting Items for Systematic Reviews and Meta-Analyses (PRISMA) statement (9). Relevant articles were identified by searching PubMed, Medline, Embase, Cochrane Library, Web of Science, ClinicalTrials.gov, CNKI, and Wanfang (the last two were Chinese literature databases) without year and language restrictions, by using the following keywords: pertuzumab or Perjeta or Rhumba 2C4; breast or mammary; randomized or randomised or randomly. The last search was updated on 17 February 2022. To identify additional articles, the reference lists of identified studies and relevant reviews were reviewed. When more than one publication was identified from the same clinical trial, we used the most recent or complete report of that trial.

We used the following selection criteria: 1) original articles reporting RCTs; 2) patients that had HER2-positive breast cancer; 3) studies that had at least two groups included: a dual anti-HER2 therapy group which is pertuzumab plus trastuzumab or T-DM1 with or without chemotherapy and a monotherapy group which is trastuzumab or T-DM1 with or without chemotherapy; 4) studies that reported at least one of the above efficacy or safety indicators; and 5) studies published in English. Studies not matching the selection criteria were excluded. Other exclusion criteria included the following: 1) repeated publications or incomplete data, 2) conference abstracts and unpublished results, and 3) phase I clinical trials.

### Data extraction and quality assessment

The following information was extracted from each included study: the first author’s name, publication year, trial names, country of origin, study design, demographics of participants, diagnosis and grading of diseases, number of patients in each group, interventions (including type, dose, and duration of anti-HER2 therapy; type of chemotherapy), follow-up time, outcomes, and other important characteristics of the study population. Data extraction was conducted independently by two investigators, and any disagreement was resolved by consensus. Risk of bias assessment was carried out using the Cochrane risk of bias tool (10). Risk of bias was rated as high/low/unclear. The quality assessment was measured using RevMan Version 5.4 (The Cochrane Collaboration, the UK).

### Statistical analysis

The pooled hazard ratio (HR) and 95% confidence interval (CI) on primary endpoints (PFS and OS) were used as the effect size of survival data. The pooled relative risk ratio (RR) and 95% CI were used to calculate the effect size of dichotomous data. Statistical heterogeneity was assessed using the *I* ^2^ and *Q*-statistic. In cases of no heterogeneity between results (*I* ^2^ < 50%; *p* > 0.1), a fixed-effect model based on Mantel–Haenszel was used; otherwise, the random-effect model was used to estimate *τ* ^2^ using DerSimonian and Laird (11, 12). A sensitivity analysis was used to assess the influence of each study on the overall results by omitting one study at a time. Potential publication bias was assessed by Begg’s test and Egger’s test. A two-sided *p*-value <0.05 was considered statistically significant. All statistical analyses were performed with Stata 12.0 (StataCorp, America).

## Results

### Search results and trial characteristics

**Figure 1:**
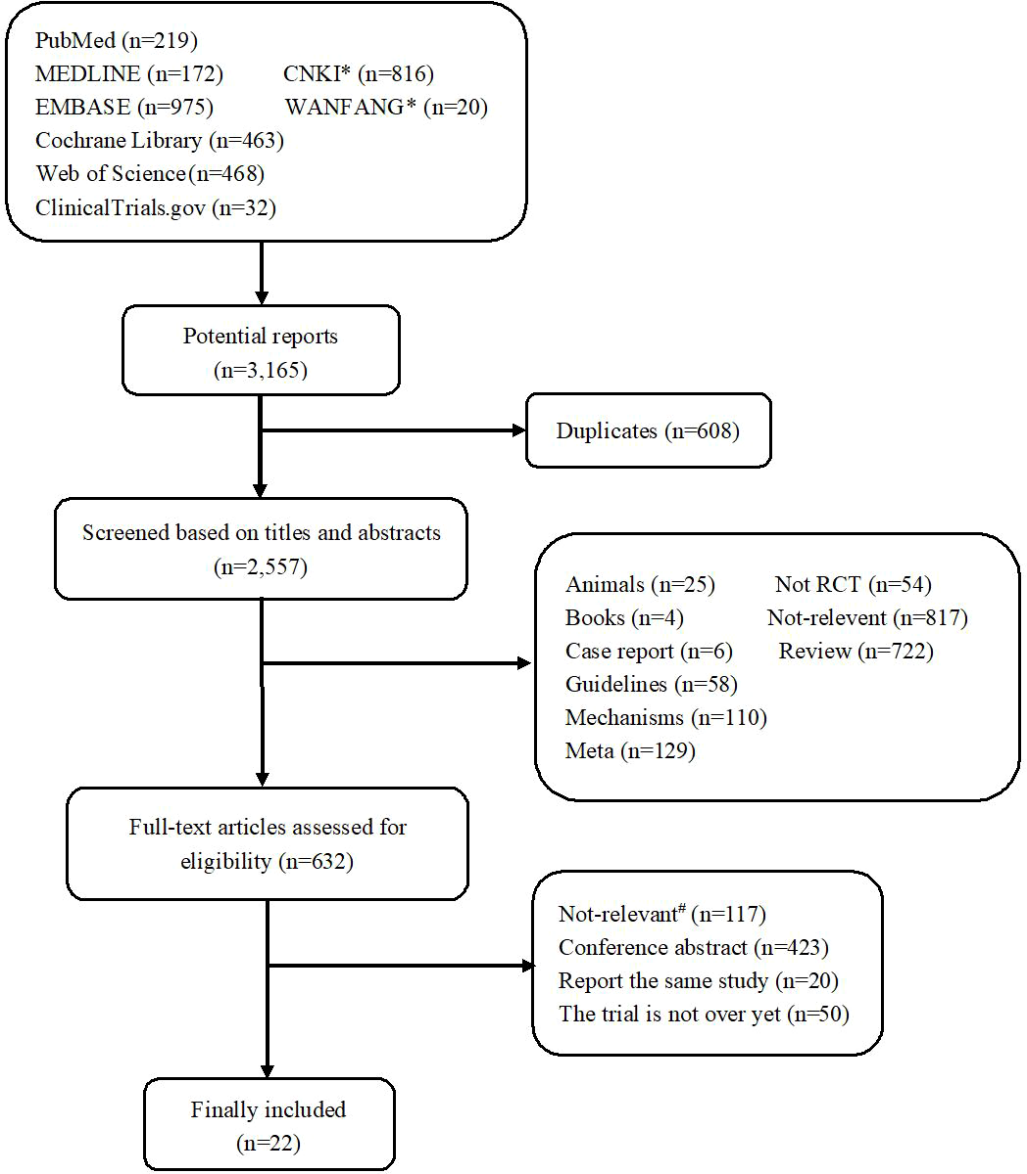
Flowchart diagram of the study selection. *CNKI and Wanfang were two Chinese literature databases. ^#^Not-relevant, including non-breast cancer (*n* = 6); both the trial group and the control group used pertuzumab (*n* = 106); the control group did not use trastuzumab (*n* = 2); phase I clinical trial (*n* = 2) and without efficacy or safety indicators (*n* = 1).

The study selection process is summarized in **Figure 1**.** A** total of 3,165 potentially relevant records were retrieved. After removing 608 duplicates, 2,557 remained for evaluation. After screening titles and abstracts, 1,925 records were excluded, including non-original research, non-relevant studies, and so on. After screening the full text, 610 records were further excluded due to the following reasons: the patients included had no breast cancer, both experimental and control groups used pertuzumab, the control group did not use trastuzumab, the studies were conference abstracts, the studies did not complete or provide results, and studies reported the same study. Thus, a total of 22 records (13–33) reporting 15 RCTs were included.

In terms of efficacy, six, one, and eight RCTs were included in the neoadjuvant, adjuvant, and advanced breast cancer therapies. One trial (28) has compared the fixed-dose combination of pertuzumab and trastuzumab for subcutaneous (SC) administration with pertuzumab IV and trastuzumab IV formulations and, thus, was excluded from the meta-analysis. The characteristics of each trial are presented in **Table 1**. Finally, 14 RCTs (8,378 patients) were included for the meta-analysis, with 4,241 patients in the dual-targeted therapy group and 4,137 in the monotherapy group. Two trials had more than three arms, but we only extracted the data related to our purpose, that is, the comparison between pertuzumab plus trastuzumab and trastuzumab alone (14, 20).

**Table 1:** Characteristics of the included studies.

Authors, year	Trial name	Region	Phase	Experimental group			Control group			Duration^b^
				Treatment	Sample size	Age^a^	Treatment	Sample size	Age^a^
Neoadjuvant setting										
Gianni, L., et al., 2012 (14, 18)	NeoSphere	International	2	T+P+D	107	–	T+D	107	50 (32–74)	12w
Buxton, M., et al., 2016 (33)	I-SPY 2 TRIAL	International	3	T+P+D-AC	150	–	T+D-AC	150	–	12w
Patel, T. A., et al., 2019 (24)	TEAL	International	2	T+P	16	57 (40–75)	T-DM1+L+/-nab-Pac	14	53 (28–70)	18w
Shao, Z., et al., 2020 (26)	PEONY	International	3	T+P+D	219	49 (24–72)	T+Placebo+D	110	49 (27–70)	12w
Tan, A. R., et al., 2021 (28)	FeDeriCa	International	3	T+P, IV	252	49 (42–58)	T+P, fdc sc	248	52 (44–59)	12w
Zhang, Q., et al., 2021 (29)	NA	China	NA	T+P+Pac	20	45.3 ± 1.3	T+Pac	20	45.3 ± 1.3	NA
Adjuvant setting										
Minckwitz, G., et al., 2017 (22)	APHINITY	International	3	T+P+FEC-D/Pac	2,400	51.7 ± 10.9	T+Placebo+FEC-D/Pac	2404	51.4 ± 10.7	52w
Metastatic setting (first line)										
Baselga, J., et al., 2012 (13, 15–17, 32)	CLEOPATRA	International	3	T+P+D	402	54.0 (27–89)	T+Placebo+D	406	54.0 (22–82)	Until^c^
Krop, I. E., et al., 2016 (19)	–	USA	2	T-DM1+P+Pac	22	54 (43–72)	T-DM1+Pac	22	50 (35–81)	Until^c^
Urruticoechea, A., et al., 2017 (21)	PHEREXA	International	3	T+P+Cap	228	54	T+Cap	224	55	Until^c^
Perez, E. A., et al., 2017 (20, 25)	MARIANNE	International	3	T-DM1+P	363	52 (27–86)	T-DM1+Placebo	367	52 (27–82)	Until^c^
Rimawi, M., et al., 2018 (23)	PERTAIN	International	2	T+P+AI	129	59 (35–87)	T+AI	129	61 (31–89)	18–24w
Xu, B., et al., 2020 (27)	PUFFIN	China	3	T+P+D	122	51 (26–74)	T+Placebo+D	121	53 (25–71)	Until^c^
Jiang, Y., et al., 2021 (31)	NA	China	NA	T+P+D	40	56.4 ± 2.4	T+D	40	56.6 ± 2.5	18w
Ma, S. Y., et al., 2021 (30)	NA	China	NA	T+P+Pac+CBP	23	43.9 ± 21.8	T+Pac+CBP	23	45.8 ± 20.9	12w

T, trastuzumab; D, docetaxel; P, pertuzumab; T-DM1, trastuzumab emtansine; Pac, paclitaxel; Cap, capecitabine; A, doxorubicin; C, cyclophosphamide; FEC, fluorouracil+epirubicin+cyclophosphamide; AI, anastrozole or letrozole; L, lapatinib; nab-pac, nab-paclitaxel; CBP, carboplatin.

a Age was expressed as median (IQR) or mean ± SD.

b Duration of anti-HER2 therapy.

c Anti-HER2 therapy was given until disease progression or unacceptable toxicity.

### Primary endpoints

**Figure 2:**
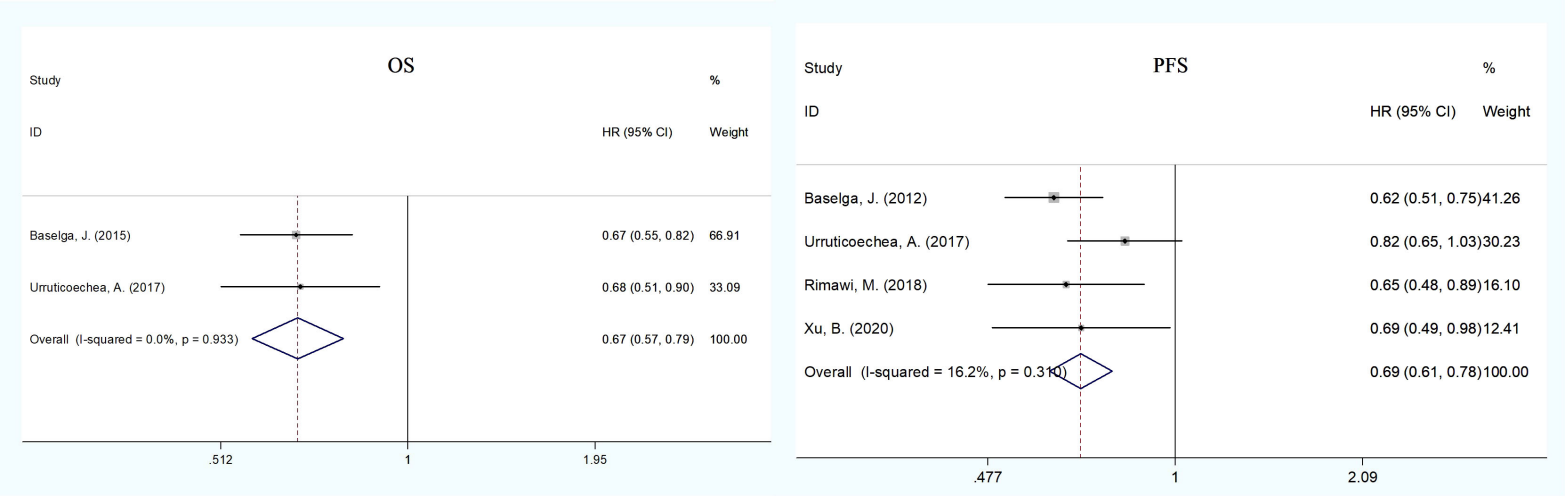
Meta-analysis of primary endpoints [overall (OS) and progression-free survival (PFS)] between the dual anti-HER2 therapy group (pertuzumabplus trastuzumab) and the monotherapy group in advanced breast cancer. The size of the squares indicates the weight of the study. Error barsrepresent 95% confidence intervals (CIs). The diamond indicates the summary odds ratio.

In the treatment of advanced breast cancer, two RCTs (13, 21, 32) reported OS, including 630 patients in the pertuzumab plus trastuzumab group and 630 in the trastuzumab group. The pooled results showed that dual anti-HER2 therapy significantly prolonged OS compared with monotherapy (HR = 0.67, 95% CI: 0.57–0.79; **Figure 2**), with no evidence of publication bias (Begg’s test, *p* = 1.000). We noted no evidence of heterogeneity across the included studies (*I* ^2^ = 0%, *p* = 0.933).

In advanced therapy, five RCTs (13, 21, 23, 27), including 881 patients of pertuzumab plus trastuzumab and 880 of trastuzumab, reported PFS data. The pooled PFS demonstrated a statistically significant improvement for patients in the dual therapy group compared to the monotherapy group (HR = 0.69, 95% CI: 0.61–0.78; **Figure 2**), with no heterogeneity across studies (*I* ^2^ = 16.2%, *p* = 0.310) and no publication bias (Egger’s test, *p* = 0.904; Begg’s test, *p* = 0.734).

### Secondary endpoints

In neoadjuvant therapy, the pCR and ORR data were reported in four (14, 26, 29, 33) and two RCTs (14, 26), respectively (**Supplementary Figure 2**). The pooled pCR and ORR had a significant absolute improvement (RR = 1.61, 95% CI: 1.30–2.01; RR = 1.11, 95% CI: 1.02–1.21) in the dual blockade group (pertuzumab plus trastuzumab) compared to the monotherapy group. In advanced therapy of pertuzumab plus trastuzumab versus trastuzumab, in evaluating the CR, PR, and ORR rates, four (13, 27, 30, 31), four (13, 27, 30, 31), and five (13, 21, 27, 30, 31) RCTs were included, respectively. The pooled PR and ORR showed a substantial benefit in the dual HER2 blocking group compared to the monotherapy group (RR = 1.23, 95% CI: 1.11–1.36; RR = 1.21, 95% CI: 1.11–1.31, respectively) with no heterogeneity among studies. There was no statistical significance in the pooled CR (RR = 1.24, 95% CI: 0.76–2.03) (**Supplementary Figure 3**). However, in advanced therapy of pertuzumab plus T-DM1 versus T-DM1, there was no statistical significance in the pooled CR, PR, and ORR (**Supplementary Figure 4**).

### Safety endpoints

Eight RCTs (13, 18, 20–22, 26, 27, 29) reported cardiotoxicities, including LVEF decline, asymptomatic left ventricular systolic dysfunction (LVSD), and heart failure (HF). The pooled HF was statistically significant in the dual HER2 blocking group compared with monotherapy (RR = 4.18, 95% CI: 1.07–16.30), whereas LVEF decline and asymptomatic LVSD did not show significant difference (**Supplementary Table 1**). We conducted an analysis of grade ≥3 AEs reported in the trials: neutropenia (eight trials), diarrhea (eight trials), febrile neutropenia (five trials), leukopenia (five trials), anemia (five trials), asthenia (four trials), fatigue (four trials), and so on (**Supplementary Table 2**). Patients receiving dual blockade of pertuzumab plus trastuzumab had a significant increase in the incidence of febrile neutropenia (RR = 1.17, 95% CI: 1.01–1.34), diarrhea (RR = 2.26, 95% CI: 1.87–2.74), and anemia (RR = 1.39, 95% CI: 1.11–1.73), whereas only diarrhea was significant in patients with pertuzumab plus T-DM1 dual therapy compared to T-DM1 monotherapy. There were no statistical differences in total grade ≥3 AEs and the other grade ≥3 AEs between the dual therapy group and the monotherapy group (**Figure 4**). The pooled analysis showed no substantial increase in the incidence of total serious AEs (RR = 1.12, 95% CI: 0.90–1.38) and death (RR = 0.83, 95% CI: 0.68–1.00) (**Figure 4** and **Supplementary Table 3**). More than half of the trials reported all-grade AEs such as diarrhea (13 trials), nausea (11 trials), rash (9 trials), neutropenia (8 trials), and alopecia (7 trials). Compared with the monotherapy group, the incidence rates of diarrhea (RR = 1.48, 95% CI: 1.30–1.69), rash (RR = 1.54, 95% CI: 1.29–1.83), and mucosal inflammation (RR = 1.45, 95% CI: 1.17–1.78) were significantly higher in the dual therapy (pertuzumab plus trastuzumab) group; however, only the incidence of rash (RR = 1.42, 95% CI: 1.07–1.87) was significant in the dual therapy of pertuzumab plus T-DM1. No other differences were observed in the other all-grade AEs (**Supplementary Table 4**).

### Subgroup analysis

**Figure 3:**
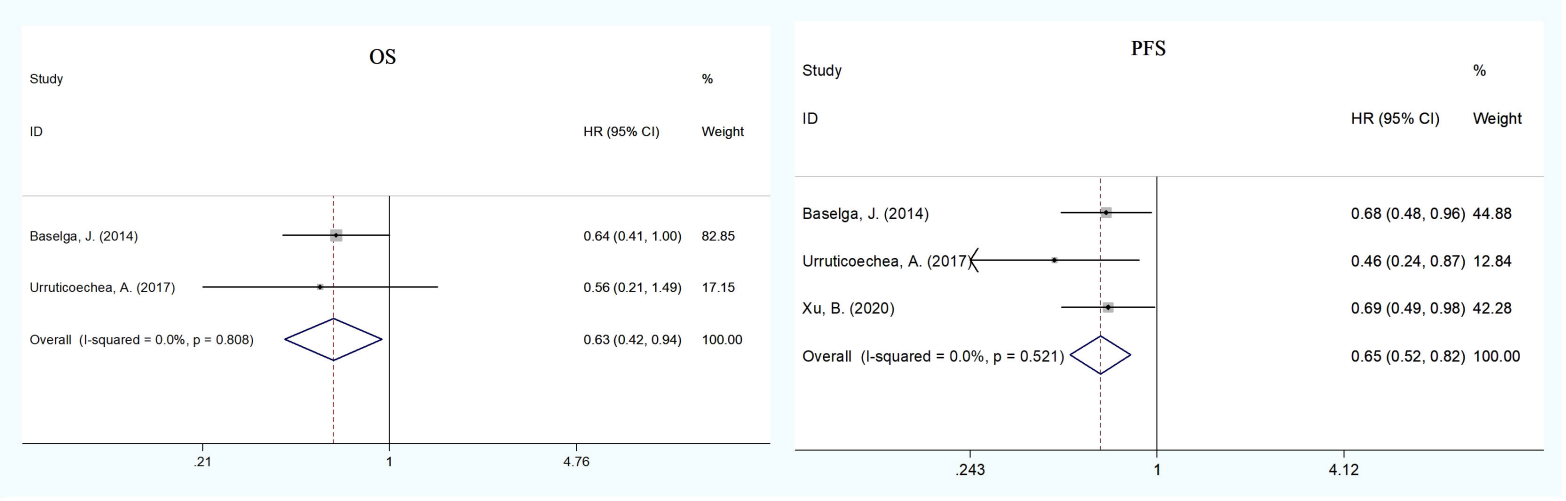
Subgroup analysis of OS and PFS between the dual anti-HER2 therapy group (pertuzumab plus trastuzumab) and the monotherapy group in advanced breast cancer in Asian patients. The size of the squares indicates the weight of the study. Error bars represent 95% confidence intervals (CIs). The diamond indicates the summary odds ratio. No evidence of publication bias was detected for OS (Begg’s test: p = 1.000) and PFS (Egger’s test: p = 0.752, Begg’s test: p = 1.000).

**Figure 4:**
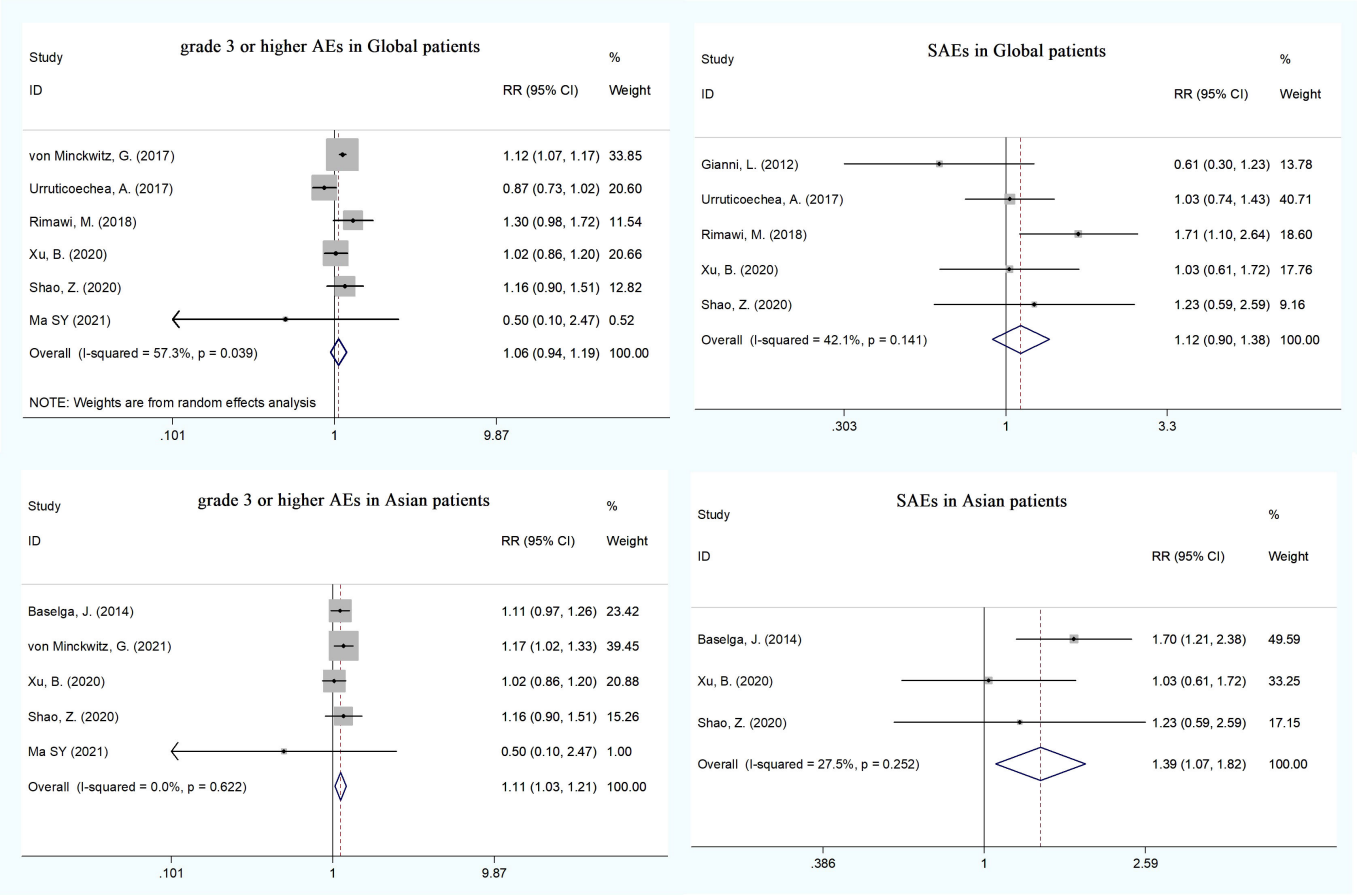
The incidence of total grade _=_3 AEs and SAEs between the dual anti-HER2 therapy group (pertuzumab plus trastuzumab) and the monotherapygroup in global and Asian patients.

To explore the effect of pertuzumab in Asian patients, we conducted a subgroup analysis and extracted data from nine publications on the treatment of advanced breast cancer (17, 20, 21, 26, 27, 29–31, 34). The pooled analysis found that the dual treatment of pertuzumab and trastuzumab resulted in an improvement in both OS and PFS compared with trastuzumab alone (HR = 0.63, 95% CI: 0.42–0.94; HR = 0.65, 95% CI: 0.52–0.82) (**Figure 3**). The incidence rates of total grade ≥3 AEs (HR = 1.11, 95% CI: 1.03–1.21) and SAEs (HR = 1.39, 95% CI: 1.07–1.82) were statistically significant in the pertuzumab plus trastuzumab group compared with the monotherapy group (**Figure 4** and **Supplementary Tables 5, 6**). For all-grade AEs, the incidence rates of diarrhea, mucosal inflammation, and infusion-related reactions were significantly higher in Asian patients treated with dual anti-HER2 therapy (**Supplementary Table 7**).

### Quality assessment and publication bias

**Figure 5:**
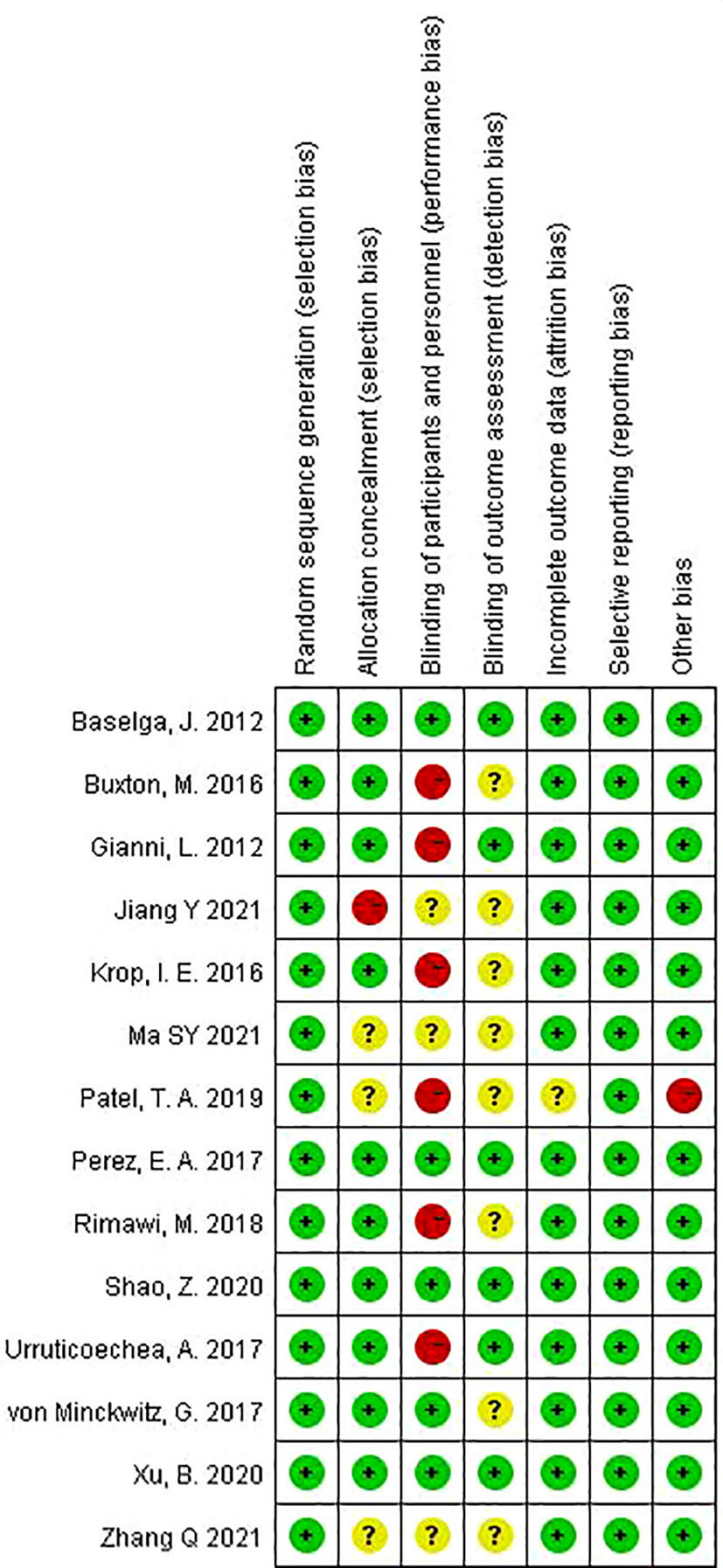
Risk of bias summary.

The risk of bias for the included trials is shown in **Figure 5** (and **Supplementary Figure 1**). Overall, the quality of the studies was satisfactory. Six trials (14, 19, 21, 23, 24, 33) were open label, that is, no blinding of the study participants and personnel. One trial (31) used a list of random numbers, which could possibly foresee assignments and, thus, introduce selection bias. One trial (24) was closed early due to superiority, with 14 patients not completing the experimental group. No evidence of significant publication bias was detected for any of the measured outcomes by Egger’s test and Begg’s test.

## Discussion

As we know, this is the first meta-analysis of RCTs focusing on the combination of pertuzumab and trastuzumab/T-DM1 therapy versus trastuzumab/T-DM1 single-agent therapy in patients with HER2-positive breast cancer. Most other studies discussed different combination regimens of dual anti-HER2 therapy (35, 36). This meta-analysis of RCTs observed the efficacy of pertuzumab plus trastuzumab with or without chemotherapy that was superior to trastuzumab monotherapy with or without chemotherapy in the treatment of advanced breast cancer, with a significant improvement in OS, PFS, PR, and ORR. In neoadjuvant therapy, the dual blockade of pertuzumab and trastuzumab has higher pCR and ORR rates than monotherapy (trastuzumab). Moreover, the total incidence of grade ≥3 AEs and SAEs did not increase in patients with dual therapy (pertuzumab plus trastuzumab) compared to monotherapy (trastuzumab). However, compared with trastuzumab, pertuzumab plus trastuzumab therapy has a higher incidence of heart failure and grade ≥3 febrile neutropenia, diarrhea, and anemia. Furthermore, in Asian patients in advanced therapy, compared to single-target therapy (trastuzumab), the double-target therapy (pertuzumab plus trastuzumab) also has a higher OS rate and PFS rate, which was consistent with the efficacy in global patients. However, the incidence of total grade ≥3 AEs and SAEs was increased significantly in Asian patients.

The HER family plays an important role in cell survival and proliferation and is implicated in oncogenesis. Overexpression of HER2 is associated with aggressive disease and poor prognosis. Both pertuzumab and trastuzumab are humanized monoclonal antibodies targeting HER2 and have proven to offer survival benefit for women with HER2-positive breast cancer. Several mechanisms have been proposed to explain the synergism of pertuzumab and trastuzumab in treating HER2-positive breast cancer, the favored theory of which was the different functions of the two antibodies (5, 37–39). Trastuzumab and T-DM1 bind to domain IV of HER2 and inhibit the homodimerization of HER2 and the downstream signaling pathways activated by the HER2 homodimer. However, pertuzumab binds HER2 in domain II, a different domain than trastuzumab, and preferentially blocks the heterodimerization of HER2 with EGFR, HER3, and HER4, and the downstream signaling pathways activated by HER2 heterodimers, which activates several intracellular signaling cascades, including cell proliferation and survival (38, 39). Therefore, the combination of the two antibodies could synergistically enhance the blocking effect of the downstream signaling, resulting in greater antitumor activity than either agent alone in preclinical studies (5). However, the German Federal Joint Committee (G-BA) and the Institute for Quality and Efficiency in Health Care (IQWiG) did not conclude that there is any additional benefit of adding pertuzumab to the neoadjuvant combination treatment of chemotherapy and trastuzumab based on the prognostic benefit, which was at that time unconfirmed (40). The combination of pertuzumab with trastuzumab and chemotherapy has been approved both by the FDA and the European Medicines Agency in the metastatic, neoadjuvant, and adjuvant settings (6, 7). Therefore, we extracted and summarized RCT studies to explore the efficacy and safety of dual blockade compared with monotherapy.

In pivotal studies of breast cancer, PFS was widely used as a primary endpoint although the choice of PFS or OS remained the subject of debate. In the CLEOPATRA trial, PFS was significantly improved with pertuzumab plus trastuzumab plus docetaxel, which was first approved in June 2012 by the FDA for the first-line treatment of HER2-positive MBC (13). After one additional year of follow-up, the OS analysis also demonstrated statistically significant and clinically meaningful survival benefit with this combination compared with trastuzumab plus docetaxel (16), which was maintained after a median follow-up of more than 8 years (41). However, the PHEREXA study did not show this consistency between PFS and OS in advanced breast cancer. It was found that adding pertuzumab to trastuzumab and capecitabine modestly increased PFS, but there was no statistical significance. Although the median OS was increased by using two anti-HER2 regimens, the statistical significance of OS cannot be claimed as a result of the hierarchical testing of OS after the primary PFS endpoint (21). In this study, after the pooling analysis, we demonstrated that dual blocking therapy could significantly prolong OS and PFS in advanced and neoadjuvant therapy in patients with HER2-positive breast cancer compared with trastuzumab single-agent therapy with or without chemotherapy. Furthermore, a subgroup analysis of the CLEOPATRA trial showed that patients experienced PFS benefit with treatment in the pertuzumab plus trastuzumab arm compared with the placebo plus trastuzumab arm in both the <65-year (HR = 0.65; 95% CI: 0.53–0.80) and >65-year groups (HR = 0.52; 95% CI: 0.31–0.86) (42). They suggested that the combined use of pertuzumab and trastuzumab should not be limited by age, though proactive management of toxicities and regular cardiac monitoring should clearly be undertaken.

pCR is an established predictor of the prognosis, and improvements in pCR appear to be associated with improvements in the prognosis to some extent (40). Combining trastuzumab and pertuzumab in neoadjuvant therapy in the NeoSphere trial resulted in a pCR rate of 45.8% and was significantly superior to neoadjuvant chemotherapy plus trastuzumab alone (29%; *p* = 0.014) (14). However, a 5-year survival analysis of this trial did not show any significant differences between the two groups (HR = 0.69; 95% CI: 0.34–1.40) (18). Two further neoadjuvant treatment trials reported that the pCR rate in patients treated with dual blockade was approximately twice as high as that in patients with trastuzumab single-agent therapy (26, 29). In our study, however, the pooled pCR was significantly increased in the dual blockade group compared to the monotherapy group in neoadjuvant therapy. A meta-analysis by Chen et al. confirmed that trastuzumab plus pertuzumab significantly improved the pCR compared to trastuzumab in neoadjuvant settings (OR = 1.33; 95% CI: 1.08–1.63; *p* = 0.006) (43). However, this study included non-RCTs, such as single-arm studies and retrospective studies, which might generate biases.

Although dual anti-HER2 therapies were associated with an efficacy benefit in HER2-positive breast cancer, they could increase the risk of cardiac toxicity in previous trials. The FDA recommendations for pertuzumab, trastuzumab, and T-DM1 limit their use to patients whose LVEF prior to treatment exceeds 50% or 55%, and the agency advises dose delay or discontinuation in the setting of LVEF decline during treatment (44). Our study showed that the combined anti-HER2 therapy increases the incidence of heart failure compared with single-agent therapy, which was not consistent with the findings from the meta-analysis, concluding that doubling up on anti-HER2 drugs did not increase cardiac toxicity compared with the use of anti-HER2 drugs individually (45). However, this study did not specify the administration of pertuzumab plus trastuzumab versus trastuzumab. Our results should be considered valid because of the included patients with an adequate cardiac function prior to therapy.

Furthermore, adverse events (any grade) such as diarrhea, rash, and mucosal inflammation which were mostly grade 1 or 2 were reported more frequently in the pertuzumab plus trastuzumab group than in the trastuzumab monotherapy group. Furthermore, a higher incidence of grade 3 or higher febrile neutropenia, diarrhea, and anemia was reported in the dual therapy group, which was consistent with the reports of the meta-analysis published by Chen et al. (43). The meta-analyses of Yu et al. (36) and Zhang et al. (46) only confirmed that dual therapy increased the incidence of grade 3–4 diarrhea because they did not collect the data of other AEs. Although our study demonstrated that dual HER2 blocking does not significantly increase the risk of total grade ≥3 AEs and total serious AEs, strict patients’ selection criteria should be adopted in future trials, and patients receiving dual regimens should be closely monitored in clinical practice. However, in the subgroup analysis of Asian patients, we found that the incidence of total grade ≥3 AEs and serious AEs was significantly higher in the dual therapy group, suggesting that clinical monitoring should be given more to Asian patients with dual-targeted therapy.

In 2002, trastuzumab (Herceptin) was initially granted regular approval by the NMPA of China, and its safety and efficacy in Chinese patients have been fully verified. In December 2018, pertuzumab was initially approved by the NMPA, and on 1 January 2020, it was included in the Chinese national reimbursement drug list (NRDL) to reduce the burden of disease. However, the data of the application of pertuzumab in Chinese patients mostly came from a subgroup analysis of international trials or bridging studies. Due to the lack of RCTs in Chinese patients, we observed the use of the combination of pertuzumab and trastuzumab therapy in global studies. This meta-analysis provides the basis for clinical practice supporting the use of the combined therapy in China. However, this meta-analysis has several limitations. First, the number of studies included was relatively small. There are some ongoing trials investigating the dual anti-HER2 therapy compared to monotherapy, and the results are yet to be released. Moreover, some studies outlined in the included studies are still in progress, and follow-up results will be used in the future analysis. Second, clinical heterogeneity does exist among trials in terms of treatment setting. Third, the calculations were based on published positive study results, and many negative results may not be published, which might generate biases.

In summary, our findings provide robust information that the combination of pertuzumab and trastuzumab with or without chemotherapy in breast cancer is warranted. The combined therapy could substantially improve the outcome of patients with HER2-positive breast cancer in both advanced and neoadjuvant therapies and was well tolerated, with no increase in total grade ≥3 AEs and serious adverse events, compared with trastuzumab monotherapy. However, in Asian patients, the incidence of total grade ≥3 AEs and SAEs was more frequent in the dual therapy group, which needs to be more closely monitored in clinical practice. Additional large-scale randomized controlled trials should be designed to further confirm the efficacy and safety of dual blocking therapy in Chinese patients with HER2-positive breast cancer.

## Data availability statement

The original contributions presented in the study are included in the article/**Supplementary Material**. Further inquiries can be directed to the corresponding author.

## Author contributions

XL and XW conceived the idea, designed the study, defined the search strategy and inclusion/exclusion criteria, and were the major contributors in writing the manuscript. XL and YF performed the literature search and the analysis. YJL, YL, and LQ contributed to the writing and editing of the manuscript. All authors contributed to the manuscript and approved the submitted version.

## Funding

The current analyses were supported by funding from Beijing Science and Technology Program (Project No. Z211100002521011).

## Conflict of interest

The authors declare that the research was conducted in the absence of any commercial or financial relationships that could be construed as a potential conflict of interest.

## Publisher’s note

All claims expressed in this article are solely those of the authors and do not necessarily represent those of their affiliated organizations, or those of the publisher, the editors and the reviewers. Any product that may be evaluated in this article, or claim that may be made by its manufacturer, is not guaranteed or endorsed by the publisher.
