# Supplementary material for: Pertuzumab combined with trastuzumab compared to trastuzumab in the treatment of HER2-positive breast cancer: A systematic review and meta-analysis of randomized controlled trials
Source: Front Oncol. 2022 Sep 28;12:894861. doi: 10.3389/fonc.2022.894861 (PMC9555237; doi:10.3389/fonc.2022.894861)
Supplement: Supplementary file 1 [file DataSheet_1.docx]

**Supplementary Material**

**Supplementary Figure 1.** Risk of bias graph.

**Supplementary Figure 2.** Meta-analysis of pathological complete response (pCR) and overall response rate (ORR) between dual anti-HER2 therapy and monotherapy in neoadjuvant therapy. The size of the squares indicates the weight of the study. Error bars represent 95% confidence intervals (CI). The diamond indicates the summary odds ratio. No evidence of publication bias was detected for pCR (Egger’s: *p*=0.898, Begg’s test: *p*=0.462) and ORR (Begg’s test: *p*=1.000).

**Supplementary Figure 3.** Meta-analysis of complete response (CR), partial response (PR) and ORR between dual anti-HER2 therapy and monotherapy in the treatment of advanced breast cancer. The size of the squares indicates the weight of the study. Error bars represent 95% confidence intervals (CI). The diamond indicates the summary odds ratio. No evidence of publication bias was detected for CR (Egger’s: *p*=0.576, Begg’s test: *p*=1.000), PR (Egger’s: *p*=0.248, Begg’s test: *p*=0.452) and ORR (Egger’s: *p*=0.273, Begg’s test: *p*=0.764)**.**

**Supplementary Table 1.** Meta-analysis of cardiotoxicities between dual anti-HER2 therapy group and monotherapy group.

**Supplementary Table 2.** Meta-analysis of grade >3 AEs between dual anti-HER2 therapy group and monotherapy group.

**Supplementary Table 3.** Meta-analysis of serious AEs between dual anti-HER2 therapy group and monotherapy group.

**Supplementary Table 4.** Meta-analysis of all-grade AEs between dual anti-HER2 therapy group and monotherapy group.

**Supplementary Figure 4.** Sensitivity analyses for the primary endpoints, including OS and PFS in advanced breast cancer therapy.

**Supplementary Figure 5.** Subgroup-analysis of CR, PR and ORR between dual anti-HER2 therapy group and monotherapy group in Asian patients in treatment of advanced breast cancer. The size of the squares indicates the weight of the study. Error bars represent 95% confidence intervals (CI). The diamond indicates the summary odds ratio. No evidence of publication bias was detected for CR (Egger’s p=0.030, Begg’s test: p=0.296), PR (Egger’s: p=0.337, Begg’s test:p=1.000) and ORR (Egger’s:p=0.334, Begg’s test p=1.000).

**Supplementary Table 5.** Subgroup-analysis of grade >3 AEs between dual anti-HER2 therapy group and monotherapy group in Asian patients.

**Supplementary Table 6.** Subgroup -analysis of serious AEs between dual anti-HER2 therapy group and monotherapy group in Asian patients.

**Supplementary Table 7.** Subgroup -analysis of all-grade AEs between dual anti-HER2 therapy group and monotherapy group in Asian patients.


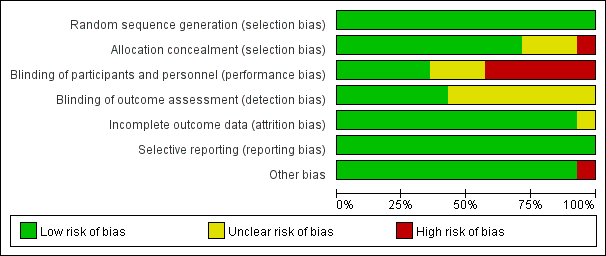


**Supplementary Figure 1. Risk of bias graph**


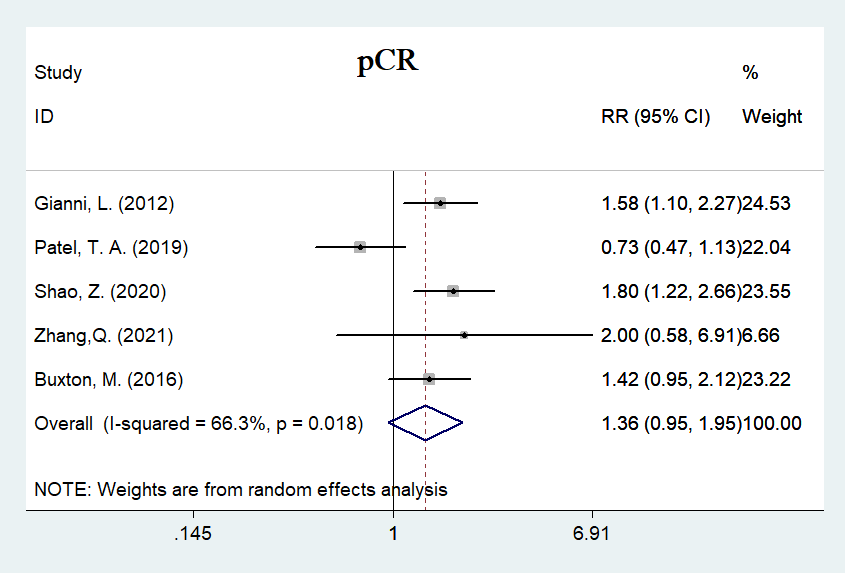

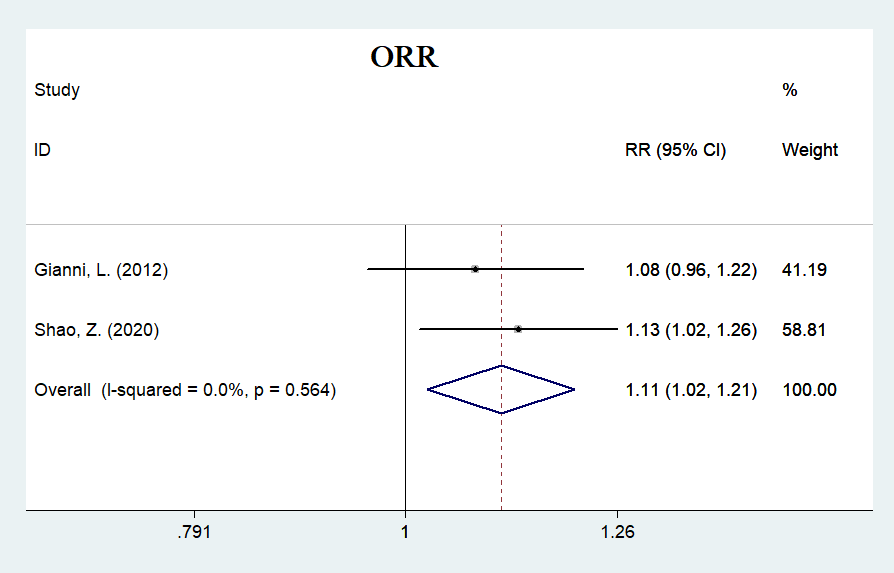


**Supplementary Figure 2.** Meta-analysis of pathological complete response (pCR) and overall response rate (ORR) between dual anti-HER2 therapy and monotherapy in neoadjuvant therapy.

The size of the squares indicates the weight of the study. Error bars represent 95% confidence intervals (CI). The diamond indicates the summary odds ratio. No evidence of publication bias was detected for pCR (Egger’s: *p*=0.898, Begg’s test: *p*=0.462) and ORR (Begg’s test: *p*=1.000).


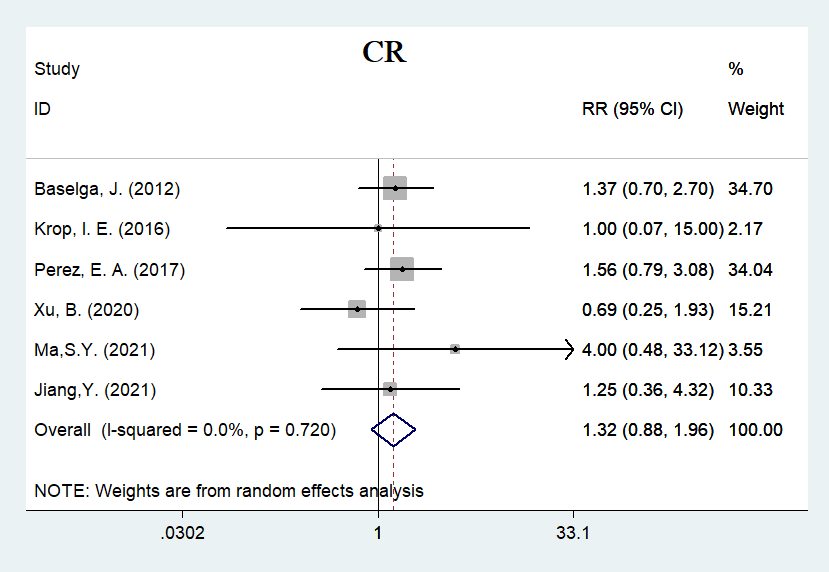

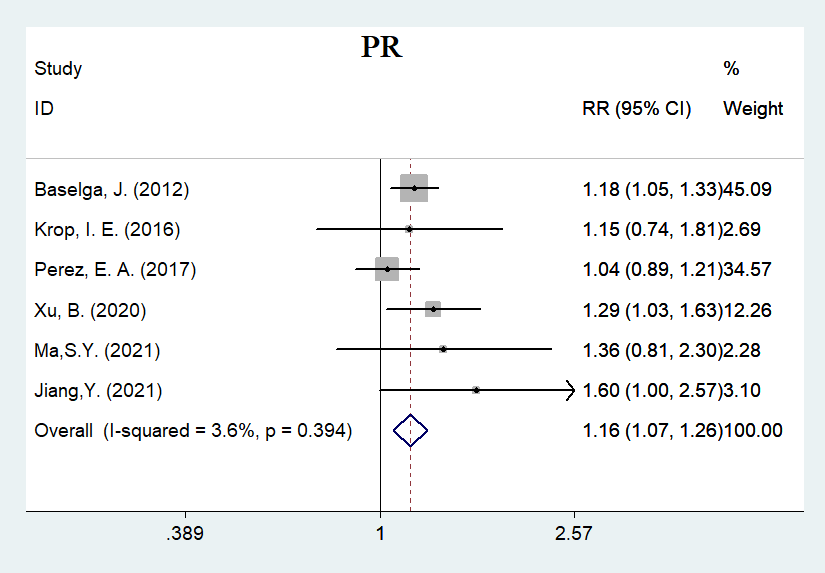

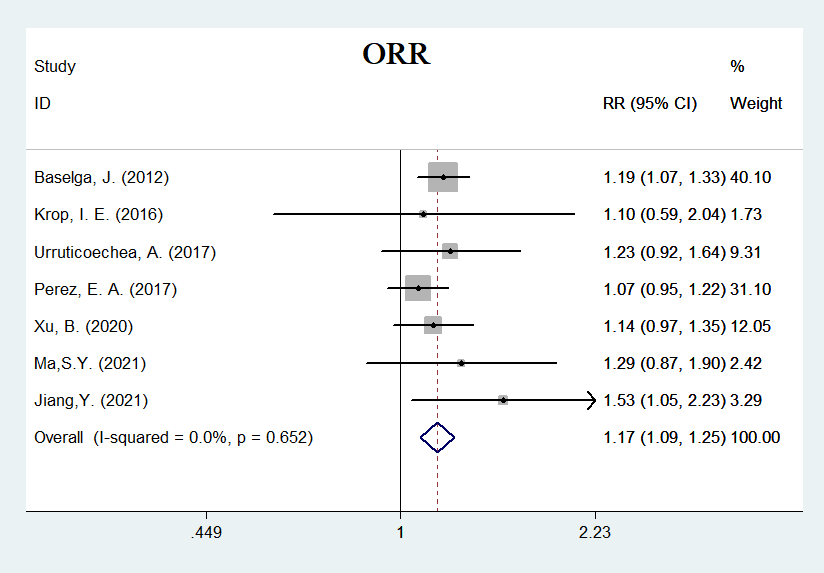


**Supplementary Figure 3.** Meta-analysis of complete response (CR), partial response (PR) and ORR between dual anti-HER2 therapy and monotherapy in the treatment of advanced breast cancer.

The size of the squares indicates the weight of the study. Error bars represent 95% confidence intervals (CI). The diamond indicates the summary odds ratio. No evidence of publication bias was detected for CR (Egger’s: *p*=0.576, Begg’s test: *p*=1.000), PR (Egger’s: *p*=0.248, Begg’s test: *p*=0.452) and ORR (Egger’s: *p*=0.273, Begg’s test: *p*=0.764)

**Supplementary Table 1.** Meta-analysis of cardiotoxicities between dual anti-HER2 therapy group and monotherapy group.

| **Cardiotoxicities** | **Trials** | **Sample size (n/N)** | | ***I^2*^*** | ***P**** | **RR (95%CI)** | **Egger’s test^#^** | **Begg’s test^#^** |
| --- | --- | --- | --- | --- | --- | --- | --- | --- |
|  |  | **Dual group** | **Monotherapy group** |  |  |  |  |  |
| LVEF decline | 5 | 37/1217 | 31/1101 | 72.5% | 0.012 | 1.94 (0.56-6.66) | 0.107 | 0.734 |
| Asymptomatic LVSD | 2 | 29/635 | 33/615 | 83.5% | 0.014 | 1.00 (0.26-3.80) | NA | 1.000 |
| HF | 3 | 10/732 | 2/614 | 0 | 0.811 | 4.18 (1.07-16.30) | NA | 1.000 |

Note: **I^2^* and *P* is statistical heterogeneity test. ^#^Egger’s test and Begg’s test are publication bias. LVEF, left ventricular ejection

fraction; LVSD, left ventricular systolic dysfunction; HF, heart failure.

**Supplementary Table 2.** Meta-analysis of grade >3 AEs between dual anti-HER2 therapy group and monotherapy group.

|  | **Trials** | **Sample size (n/N)** | | ***I^2*^*** | ***P**** | **RR (95%CI)** | **Egger’s test^#^** | **Begg’s test^#^** |
| --- | --- | --- | --- | --- | --- | --- | --- | --- |
| **Grade >3 AEs** |  | **Dual group** | **Monothreapy group** |  |  |  |  |  |
| Total | 7 | 2063/3448 | 1854/3361 | 52.4% | 0.050 | 1.05 (0.96-1.16) | 0.405 | 1.000 |
| Neutropenia | 8 | 805/3939 | 763/3842 | 24.0% | 0.238 | 1.00 (0.92-1.09) | 0.079 | 0.386 |
| Febrile neutropenia | 5 | 361/3127 | 313/3153 | 33.6% | 0.198 | 1.17 (1.01-1.34) | 0.555 | 0.806 |
| Leukopenia | 5 | 161/981 | 150/858 | 19.7% | 0.289 | 0.92 (0.76-1.11) | 0.248 | 0.462 |
| Anemia | 5 | 201/3286 | 147/3309 | 0 | 0.490 | 1.38 (1.12-1.70) | 0.876 | 1.000 |
| Diarrhea | 8 | 331/3743 | 143/3754 | 8.9% | 0.361 | 2.31 (1.91-2.80) | 0.814 | 0.266 |
| Nausea | 2 | 7/250 | 8/240 | 0 | 0.894 | 0.84 (0.31-2.28) | NA | 1.000 |
| Asthenia | 4 | 16/663 | 10/650 | 0 | 0.600 | 1.54 (0.72-3.30) | 0.421 | 1.000 |
| Fatigue | 4 | 14/572 | 17/557 | 0 | 0.569 | 0.81 (0.42-1.58) | 0.398 | 0.734 |
| Neuropathy peripheral | 3 | 19/556 | 9/543 | 0 | 0.628 | 2.02 (0.95-4.29) | 0.165 | 0.296 |
| Rash | 3 | 4/457 | 3/445 | 0 | 0.464 | 1.23 (0.33-4.54) | 0.732 | 1.000 |
| Hypertension | 3 | 39/901 | 37/881 | 0 | 0.908 | 1.03 (0.67-1.60) | 0.884 | 1.000 |
| ALT increased | 3 | 25/595 | 19/588 | 57.2% | 0.097 | 1.28 (0.20-8.12) | 0.573 | 1.000 |
| Hypokalaemia | 2 | 4/143 | 1/138 | 7% | 0.300 | 2.79 (0.46-16.91) | NA | 1.000 |
| Mucosal inflammation | 3 | 5/271 | 1/266 | 0 | 0.627 | 2.96 (0.60-14.47) | 0.743 | 1.000 |

Note: **I^2^* and *P* is statistical heterogeneity test. ^#^Egger’s test and Begg’s test are publication bias.

**Supplementary Table 3.** Meta-analysis of serious AEs between dual anti-HER2 therapy group and monotherapy group.

| **SAE** | **Trials** | **Sample size (n/N)** | | ***I^2^*** | ***P**** | **RR (95%CI)** | **Egger’s test** | **Begg’s test** |
| --- | --- | --- | --- | --- | --- | --- | --- | --- |
|  |  | **Dual group** | **Monotherapy group** |  |  |  |  |  |
| Total | 5 | 155/802 | 126/679 | 42.1% | 0.141 | 1.12 (0.90-1.38) | 0.793 | 1.000 |
| Death | 7 | 300/3809 | 339/3724 | 0 | 0.600 | 0.88 (0.77-1.01) | 0.344 | 0.548 |

Note: * *I^2^* and *P* is statistical heterogeneity test. # Egger’s test and Begg’s test are publication bias.

**Supplementary Table 4.** Meta-analysis of all-grade AEs between dual anti-HER2 therapy group and monotherapy group.

| **All-grade AEs** | **trials** | **Sample size (n/N)** | | ***I^2*^*** | ***P**** | **RR (95%CI)** | **Egger’s test^#^** | **Begg’s test^#^** |
| --- | --- | --- | --- | --- | --- | --- | --- | --- |
|  |  | **Dual group** | **Monotherapy group** |  |  |  |  |  |
| Blood and lymphatic system disorders | | |  |  |  |  |  |  |
| Neutropenia | 8 | 521/1486 | 479/1349 | 23.2% | 0.245 | 0.97 (0.88-1.06) | 0.101 | 0.536 |
| Leukopenia | 2 | 181/340 | 129/230 | 0 | 0.699 | 1.04 (0.90-1.21) | NA | 1.000 |
| Thrombocytopenia | 2 | 3/38 | 4/36 | 0 | 0.485 | 0.76 (0.20-2.79) | NA | 1.000 |
| Anaemia | 4 | 147/489 | 107/376 | 0 | 0.480 | 1.09 (0.90-1.34) | 0.354 | 0.734 |
| Gastrointestinal disorders | | |  |  |  |  |  |  |
| Diarrhea | 13 | 2574/4060 | 1637/3961 | 63.9% | 0.001 | 1.56 (1.49-1.62) | 0.981 | 0.760 |
| Nausea | 11 | 610/1656 | 555/1516 | 0 | 0.766 | 1.04 (0.95-1.13) | 0.283 | 0.876 |
| Vomiting | 6 | 297/1153 | 239/1131 | 0 | 0.702 | 1.22 (1.05-1.41) | 0.783 | 1.000 |
| Constipation | 5 | 137/831 | 186/823 | 14.5% | 0.322 | 0.73 (0.60-0.89) | 0.463 | 0.806 |
| General disorders and administration site conditions | | | |  |  |  |  |  |
| Asthenia | 4 | 192/763 | 190/748 | 15.8% | 0.313 | 0.99 (0.83-1.18) | 0.087 | 1.000 |
| Fatigue | 6 | 338/942 | 324/936 | 0 | 0.833 | 1.04 (0.92-1.17) | 0.392 | 0.133 |
| Peripheral edema | 4 | 166/1022 | 193/1002 | 49.0% | 0.117 | 0.84 (0.70-1.01) | 0.704 | 0.734 |
| Pyrexia | 3 | 175/706 | 129/591 | 0 | 0.708 | 1.23 (1.01-1.50) | 0.098 | 1.000 |
| Nervous system disorders | | |  |  |  |  |  |  |
| Neuropathy peripheral | 3 | 82/404 | 69/397 | 62.6% | 0.069 | 1.01 (0.62-1.64) | 0.870 | 1.000 |
| Headache | 3 | 152/600 | 142/592 | 0 | 0.443 | 1.06 (0.87-1.28) | 0.566 | 1.000 |
| Skin and subcutaneous tissue disorders | | |  |  |  |  |  |  |
| Rash | 9 | 353/1435 | 230/1403 | 22.5% | 0.243 | 1.50 (1.30-1.74) | 0.214 | 0.118 |
| Mucosal inflammation | 5 | 173/674 | 116/660 | 0 | 0.500 | 1.46 (1.19-1.80) | 0.359 | 0.806 |
| Alopecia | 7 | 553/1369 | 474/1241 | 0 | 0.477 | 1.04 (0.95-1.13) | 0.107 | 0.133 |
| Nail discoloration | 2 | 12/138 | 16/134 | 0 | 0.547 | 0.74 (0.37-1.48) | NA | 1.000 |
| Respiratory, thoracic, and mediastinal disorders | | | |  |  |  |  |  |
| Cough | 3 | 107/507 | 90/509 | 0 | 0.550 | 1.20 (0.93-1.54) | 0.573 | 1.000 |
| Epistaxis | 3 | 138/404 | 119/397 | 46.2% | 0.156 | 1.14 (0.93-1.40) | 0.741 | 1.000 |
| Upper respiratory tract infection | 2 | 45/340 | 20/230 | 67.8% | 0.078 | 1.46 (0.56-3.77) | NA | 1.000 |
| Dyspnea | 2 | 26/149 | 15/146 | 0 | 0.562 | 1.70 (0.94-3.07) | NA | 1.000 |
| Musculoskeletal and connective tissue disorders | | | |  |  |  |  |  |
| Myalgia | 5 | 99/534 | 97/527 | 0 | 0.899 | 1.01 (0.79-1.30) | 0.009 | 0.027 |
| Arthralgia | 3 | 113/515 | 118/507 | 17.4% | 0.298 | 0.94 (0.75-1.18) | 0.916 | 1.000 |
| Others |  |  |  |  |  |  |  |  |
| ALT increased | 2 | 84/304 | 76/230 | 26.2% | 0.244 | 0.80 (0.61-1.04) | NA | 1.000 |
| Anxiety | 2 | 13/143 | 5/138 | 0 | 0.942 | 2.37 (0.91-6.22) | NA | 1.000 |
| Decreased appetite | 5 | 245/1041 | 218/1030 | 0 | 0.542 | 1.11 (0.95-1.30) | 0.258 | 0.462 |
| Hypersensitivity | 2 | 8/249 | 1/244 | 48.8% | 0.162 | 5.55 (0.98-31.33) | NA | 1.000 |

Note: * *I^2^* and *P* is statistical heterogeneity test. # Egger’s test and Begg’s test are publication bias.


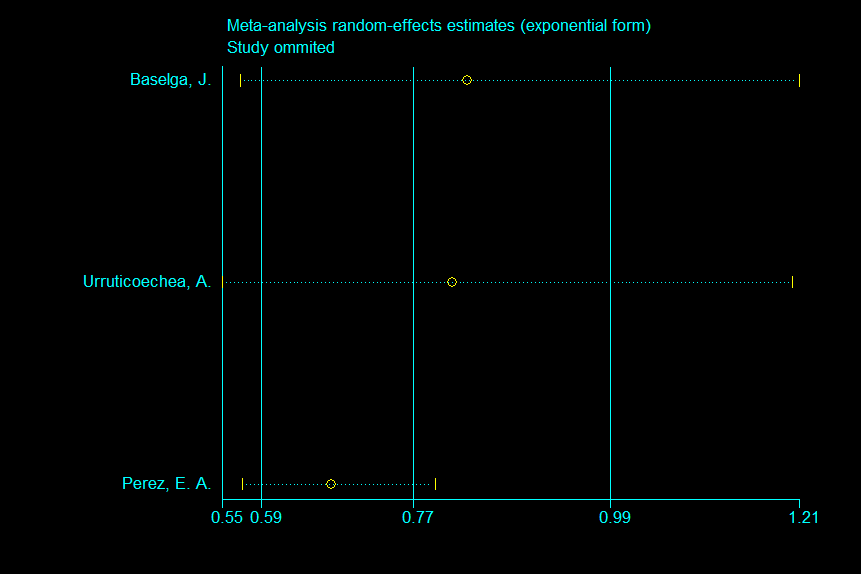

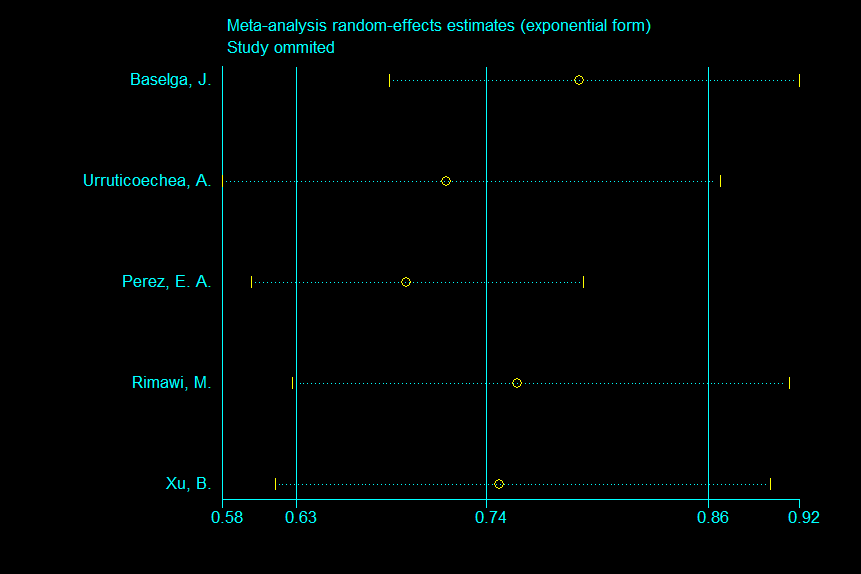


**Supplementary Figure 4.** Sensitivity analyses for the primary endpoints, including OS (left) and PFS (right) in advanced breast cancer therapy.


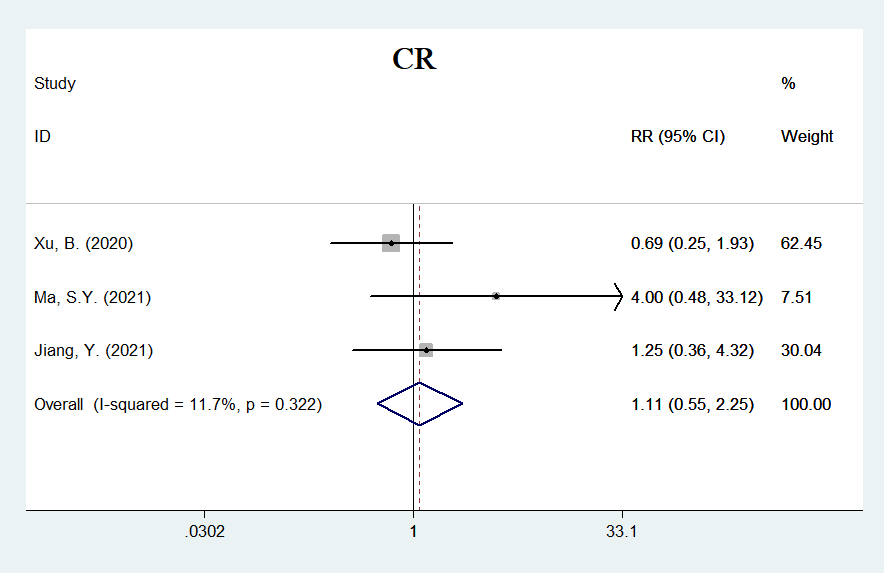

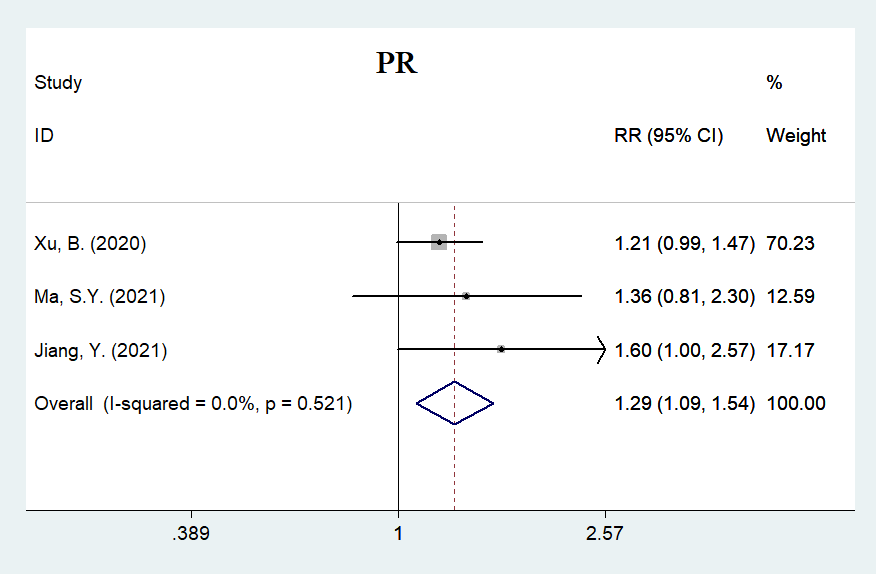

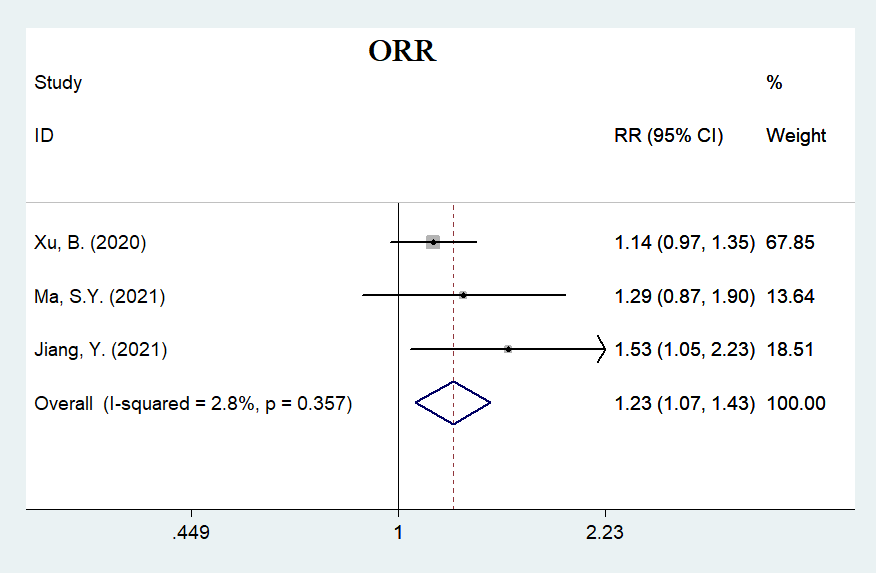


**Supplementary Figure 5.** Subgroup-analysis of CR, PR and ORR between dual anti-HER2 therapy group and monotherapy group in Asian patients in treatment of advanced breast cancer.

The size of the squares indicates the weight of the study. Error bars represent 95% confidence intervals (CI). The diamond indicates the summary odds ratio. No evidence of publication bias was detected for CR (Egger’s p=0.030, Begg’s test: p=0.296), PR (Egger’s: p=0.337, Begg’s test:p=1.000) and ORR (Egger’s:p=0.334, Begg’s test p=1.000).

**Supplementary Table 5.** Subgroup-analysis of grade >3 AEs between dual anti-HER2 therapy group and monotherapy group in Asian patients.

|  | **Trials** | **Sample size (n/N)** | | ***I^2^**** | ***P**** | **RR (95%CI)** | **Egger’s test^#^** | **Begg’s test^#^** |
| --- | --- | --- | --- | --- | --- | --- | --- | --- |
| **Grade>3 AEs** |  | **Dual group** | **Monotherapy group** |  |  |  |  |  |
| Total | 5 | 478/762 | 389/665 | 0 | 0.622 | 1.12 (1.03-1.21) | 0.453 | 0.806 |
| Neutropenia | 3 | 204/614 | 159/514 | 0 | 0.532 | 1.04 (0.88-1.23) | 0.354 | 1.000 |
| Febrile neutropenia | 2 | 23/396 | 15/404 | 65.3% | 0.090 | 1.36 (0.42-4.39) | NA | 1.000 |
| Leukopenia | 3 | 137/614 | 110/514 | 0 | 0.975 | 1.05 (0.85-1.29) | 0.871 | 1.000 |
| Diarrhea | 2 | 19/396 | 10/404 | 0 | 0.783 | 1.94 (0.91-4.12) | NA | 1.000 |

Note: **I^2^* and *P* is statistical heterogeneity test. ^#^Egger’s test and Begg’s test are publication bias.

**Supplementary Table 6.** Subgroup -analysis of serious AEs between dual anti-HER2 therapy group and monotherapy group in Asian patients.

|  | | **Trials** | | **Sample size (n/N)** | | ***I^2^**** | ***P**** | **RR (95%CI)** | **Egger’s test^#^** | **Begg’s test^#^** |
| --- | --- | --- | --- | --- | --- | --- | --- | --- | --- | --- |
| **SAE** | |  |  | **Dual group** | **Monotherapy group** |  |  |  |  |  |
| Total | | 3 | 104/465 | 67/358 | 27.5% | 0.252 | 1.39 (1.07-1.82) | 0.450 | 1.000 | |
| Death | | 4 | 17/739 | 14/642 | 0 | 0.663 | 1.15 (0.59-2.22) | 0.224 | 0.734 | |

Note: **I^2^* and *P* is statistical heterogeneity test. ^#^Egger’s test and Begg’s test are publication bias.

**Supplementary Table 7.** Subgroup -analysis of all-grade AEs between dual anti-HER2 therapy group and monotherapy group in Asian patients.

|  | **Trials** | **Sample size (n/N)** | | ***I^2^*(%)^*^** | ***P**** | **RR (95%CI)** | **Egger’s test^#^** | **Begg’s test^#^** |
| --- | --- | --- | --- | --- | --- | --- | --- | --- |
| **All grade AEs** |  | **Dual group** | **Monotherapy group** |  |  |  |  |  |
| Blood and lymphatic system disorders | | | |  |  |  |  |  |
| Neutropenia | 3 | 265/465 | 207/358 | 0 | 0.890 | 1.03 (0.92-1.16) | 0.210 | 0.296 |
| Leukopenia | 2 | 181/340 | 129/230 | 0 | 0.699 | 1.04 (0.90-1.21) | NA | 1.000 |
| Anaemia | 2 | 117/340 | 87/230 | 0 | 0.354 | 1.02 (0.82-1.26) | NA | 1.000 |
| Gastrointestinal disorders | | | |  |  |  |  |  |
| Diarrhea | 6 | 241/548 | 121/441 | 50 | 0.075 | 1.69 (1.43-2.01) | 0.994 | 1.000 |
| Nausea | 5 | 117/508 | 97/401 | 0 | 0.966 | 0.99 (0.79-1.25) | 0.982 | 1.000 |
| Vomiting | 3 | 47/270 | 63/271 | 75.5 | 0.017 | 0.76 (0.28-2.05) | 0.490 | 1.000 |
| Constipation | 2 | 33/148 | 43/151 | 0 | 0.396 | 0.79 (0.54-1.15) | NA | 1.000 |
| General disorders and administration site conditions | | | | | |  |  |  |
| Fatigue | 3 | 60/168 | 72/171 | 0 | 0.834 | 0.85 (0.65-1.11) | 0.127 | 0.296 |
| Peripheral edema | 2 | 50/247 | 61/248 | 45.6 | 0.175 | 0.83 (0.60-1.14) | NA | 1.000 |
| Pyrexia | 2 | 57/340 | 29/230 | 0 | 0.998 | 1.42 (0.94-2.16) | NA | 1.000 |
| Skin and subcutaneous tissue disorders | | | | |  |  |  |  |
| Rash | 3 | 92/287 | 75/288 | 28.5 | 0.247 | 1.24 (0.97-1.58) | 0.13 | 1.000 |
| Mucosal inflammation | 2 | 73/247 | 35/248 | 0 | 0.843 | 2.11 (1.47-3.02) | NA | 1.000 |
| Alopecia | 3 | 230/465 | 178/358 | 30.9 | 0.235 | 1.00 (0.87-1.15) | 0.029 | 0.296 |
| Nail discoloration | 2 | 61/247 | 67/248 | 0 | 0.602 | 0.92 (0.70-1.23) | NA | 1.000 |
| Respiratory, thoracic, and mediastinal disorders | | | |  |  |  |  |  |
| Upper respiratory tract infection | 3 | 45/340 | 20/230 | 38.0 | 0.200 | 1.38 (0.99-1.92) | 0.44 | 1.000 |
| Musculoskeletal and connective tissue disorders | | | |  |  |  |  |  |
| Myalgia | 2 | 60/148 | 58/151 | 0 | 0.798 | 1.06 (0.80-1.40) | NA | 1.000 |
| Others |  |  |  |  |  |  |  |  |
| ALT increased | 2 | 84/340 | 76/230 | 26.2 | 0.244 | 0.80 (0.61-1.04) | NA | 1.000 |
| Decreased appetite | 2 | 82/247 | 66/248 | 0 | 0.540 | 1.26 (0.98-1.62) | NA | 1.000 |
| Infusion-related reactions | 2 | 86/340 | 37/230 | 52.6 | 0.147 | 1.74 (1.00-3.01) | NA | 1.000 |

Note: **I^2^* and *P* is statistical heterogeneity test. ^#^Egger’s test and Begg’s test are publication bias.
